# Dual mode of cell death upon the photo-irradiation of a Ru^II^ polypyridyl complex in interphase or mitosis†
†Electronic supplementary information (ESI) available. See DOI: 10.1039/c6sc00387g


**DOI:** 10.1039/c6sc00387g

**Published:** 2016-06-01

**Authors:** Vanessa Pierroz, Riccardo Rubbiani, Christian Gentili, Malay Patra, Cristina Mari, Gilles Gasser, Stefano Ferrari

**Affiliations:** a Institute of Molecular Cancer Research , University of Zurich , Winterthurerstrasse 190 , CH-8057 Zurich , Switzerland . Email: sferrari@imcr.uzh.ch ; http://www.imcr.uzh.ch/research/Ferrari.html ; Fax: +41 44 635 3484 ; Tel: +41 44 635 3471; b Department of Chemistry , University of Zurich , Winterthurerstrasse 190 , CH-8057 Zurich , Switzerland . Email: gilles.gasser@chem.uzh.ch ; http://www.gassergroup.com ; Fax: +41 44 635 6803 ; Tel: +41 44 635 4630

## Abstract

Photodynamic therapy (PDT) is an attractive, complementary medical technique to chemotherapy. Among the different photosensitizers (PSs) employed, Ru(ii) polypyridyl complexes were found to be valid substitutes to porphyrin-based or phthalocyanine-based PSs. Here, we confirm that one such complex, namely [Ru(bipy)_2_-dppz-7-methoxy][PF_6_]_2_ (Ru65), which localizes in the nucleus of various cancer and normal cells, displays cytotoxicity only upon UV-A irradiation. Importantly, we disclose the molecular mechanism of the UV-A mediated cytotoxic action of Ru65. We demonstrate that Ru65 intercalates in DNA and, upon light irradiation, promotes guanine oxidation, resulting in nicks in the double helix. We confirm this mechanism of action in living cells, showing that the UV-A irradiation of cells loaded with Ru65 results in a transient DNA damage response and cell death. Strikingly, the photo-irradiation of Ru65 triggered distinct mechanisms of cell death in interphase or mitotic cells. The former underwent cell cycle arrest at the G2/M phase and massive cytoplasmic vacuolation, which was paralleled by an unfolded-protein stress response, resulting in a reduction of viability and cell death through a paraptosis-like mechanism. On the other hand, the UV-A irradiation of Ru65 in cells synchronized by G2/M block-release with a selective CDK1 inhibitor led to blocking mitotic entry and rapid cell death through classic apoptotic pathways. Importantly, targeting mitotic cells with Ru65 allowed increasing its photo-toxicity by a factor of 3.6. Overall, our findings show that the use of a combination of a cell cycle inhibitor and a PS targeting the nucleus could open up new avenues in PDT.

## Abstract 2

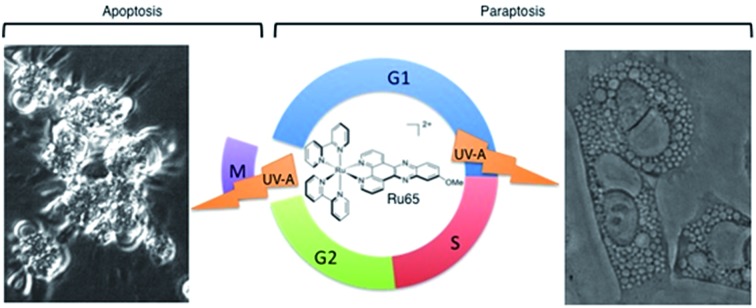
The modes of action of a Ru(ii) polypyridyl complex upon light irradiation are described.

## Introduction

Cancer therapy is still largely based on the use of DNA damaging agents, despite evident drawbacks represented by their intrinsic genotoxic potential.^1^,^2^ In recent years, the rational design of molecules targeting oncogenic pathways that are hyper-functional in cancer cells or to which cancer cells become addicted^1^,^3^ has provided an alternative approach to the use of “dirty” drugs. The well-documented adaptability of cancer cells to external insults, however, which is exemplified by the case of BCR-ABL where point mutations in the binding site for inhibitory molecules like Gleevec render the kinase resistant to first generation compounds,^4^ continuously demands the implementation of novel strategies.

The use of light to activate drugs has gained increasing attention as a promising alternative to current protocols deployed to treat a number of pathologies. Among the different medical techniques relying on light activation (*e.g.* photo-thermal therapy, photoactivated chemotherapy, *etc.*), photodynamic therapy (PDT) has been undoubtedly the most successful method reported so far. Initially used in the therapy of macular degeneration and bacterial infections, PDT has been increasingly successful in the treatment of a variety of cancers, including those of the skin or, supported by the use of optic fibers to reach cavities, in targeting cancers of the lung, esophagus, and prostate.^5^,^6^ More specifically, PDT relies on the irradiation of a photosensitizer (PS) by light at a defined wavelength, resulting in the production of reactive oxygen species (ROS), mainly consisting of singlet oxygen (^1^O_2_),^7^ which has an estimated half-life of 40 ns in a biological environment.^8^ ROS rapidly react with biomolecules in close proximity to the PS, impairing metabolic functions and ultimately leading to cell death. The advantage of PDT over conventional chemotherapeutic treatments of solid tumors lies in its decreased systemic toxicity, since the generation of ROS can be guided by light at defined locations as opposed to the mere uptake of an active drug in all cells. PDT appears also, in certain cases, to be a better alternative to chemotherapy when used in combination with surgery.^9^ Indeed, the local application of PDT upon surgical removal of portions of tissues or organs allows targeting the tumor while preserving healthy tissue, not last to the benefit of the aesthetic. Nonetheless, currently approved PSs based on porphyrin and phthalocyanine suffer from drawbacks inherent to their molecular structure, such as low solubility in water and prolonged photosensitivity for the patients.^10^ Good PSs should ideally display high solubility in water, good phototoxic dark/light index (PI), good ^1^O_2_ quantum yield upon activation with non-harmful light, and little or no photosensitivity for the patient.^11^

In recent years, ruthenium complexes have entered the arena of anticancer drug candidates.^12^–^18^ Interestingly, the clarification of *in vitro* and *in vivo* anti-proliferative and anti-metastatic properties of heterocyclic Ru(iii)-complexes (KP1039 and NAMI-A) led to the initiation of clinical trials.^12^,^19^ In addition, a Ru(ii) polypyridyl complex acting as a PS in the PDT of bladder cancer will soon be promoted to clinical trials (; http://theralase.com/pressrelease/theralase-signs-clinical-research-agreement-university-health-network/).^20^ A compound based on the same chemical structure, namely a substitutionally inert Ru(ii) polypyridyl complex [Ru(bipy)_2_-dppz-7-methoxy][PF_6_]_2_ (Ru65 hereafter), that acts as excellent PDT agent was recently described by our laboratory.^21^ Ru65, whose phosphorescence can be turned on upon intercalation in a hydrophobic environment, showed nuclear localization in living cells and the ability to nick plasmid DNA *in vitro*, suggesting that it intercalates between bases and damages DNA upon irradiation-induced singlet oxygen (^1^O_2_) production.^21^ This finding is of interest since, to date, the nucleus has been largely neglected as a potential targeting compartment,^22^ and instead the majority of current PDT PS target the mitochondria, endoplasmic reticulum (ER), golgi apparatus, plasma or cytosolic membranes, cytosol or lysosomes.^23^,^24^ However, it is worth remarking that our Ru(ii) polypyridyl complex still displays a few drawbacks compared to current PSs on the market, including low absorbance at short wavelength in the visible (20 000 M^–1^ cm^–1^ at 440 nm). Moreover, our experiments have been performed upon UV light irradiation, which is not the ideal setting for certain PDT treatments.

In the present study, we set out to characterize the mechanism of action of Ru65. We observed that low-dose UV-A irradiation of DNA intercalated Ru65 triggered a transient DNA damage response. This was followed by a sustained arrest at the S and G2/M phases of the cell cycle and accompanied by extensive cytoplasmic vacuolization, an unfolded-protein stress response, loss of viability, and cell death. We also report that, upon light irradiation, cells treated with Ru65 at the G2/M transition of the cell cycle could not enter mitosis and rapidly died. These findings provide the grounds for future studies on the use of a combination of cell cycle inhibitors and nucleus-targeting PS in PDT.

## Results

### Photo-irradiation of Ru65 causes oxidative damage to DNA bases

**Fig. 1:**
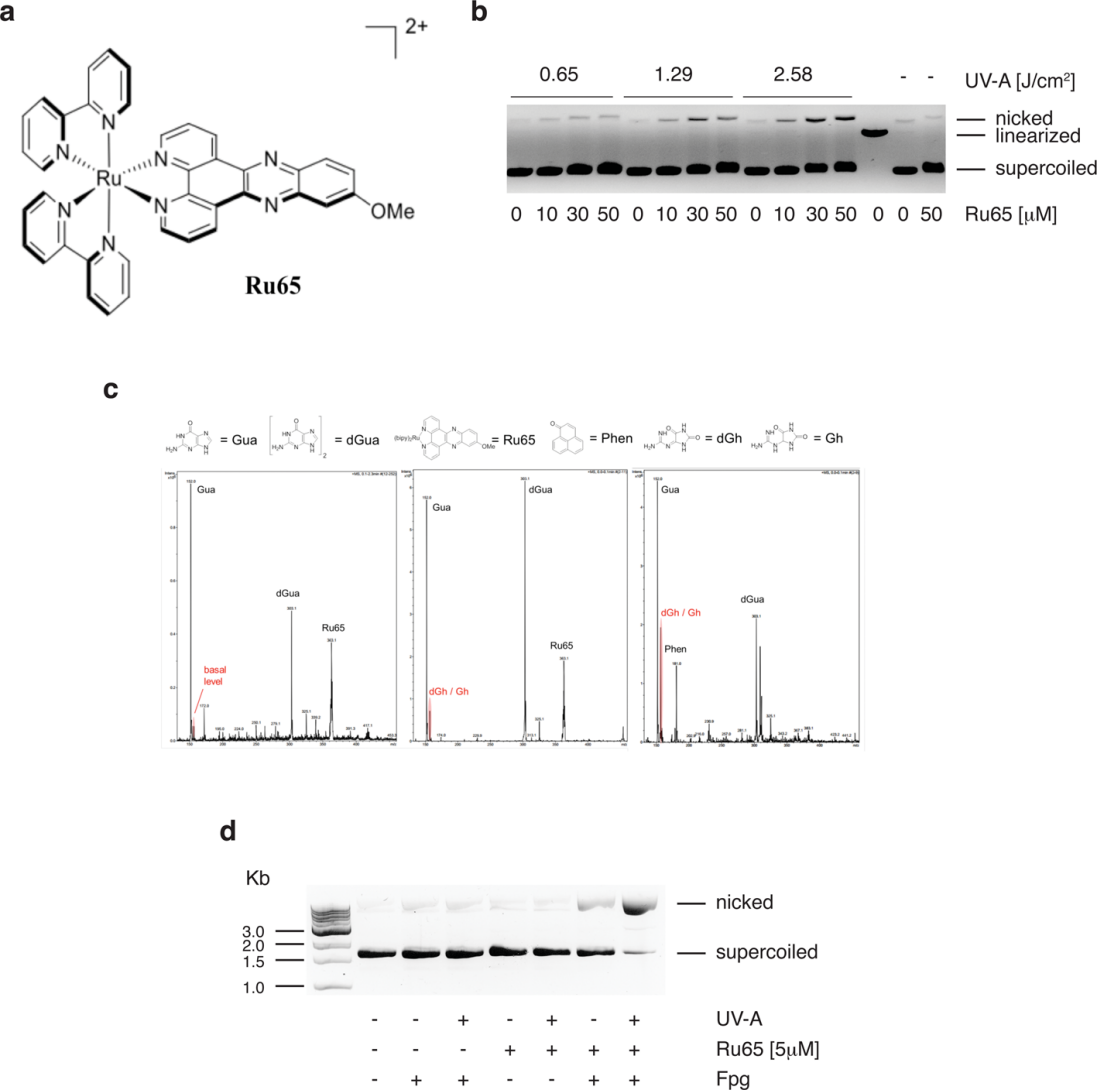
Photo-irradiation of Ru65 causes base damage in plasmid DNA. (a) Structure of [Ru(bipy)_2_-dppz-7-methoxy][PF_6_]_2_ (Ru65). (b) The plasmid pUC18 (150 ng) was incubated with the indicated amounts of Ru65 for 30 min and either UV-A irradiated or not. Products were resolved on a 1% agarose gel in the presence of EtBr. A linearized form of the plasmid was obtained by EcoRI digestion. The positions of the supercoiled, linearized, and nicked plasmid are indicated. (c) Detection of guanine oxidation sub-products by mass spectrometry. Left panel: non-irradiated guanine and Ru65; middle panel: guanine and Ru65 treated with UV-A; right panel: guanine and phenalenone (100 μM) used as positive control. Gua: guanine; dGua: deoxy-guanine; dGh: deoxy-guanidinohydantoin; Gh: guanidinohydantoin. (d) Purine oxidation was assessed by treating pUC18 (150 ng) with Ru65 and UV-A as indicated, followed by incubation with 0.2 U of Fpg for 1 h at 37 °C. The products were resolved on a 1% agarose gel in the presence of EtBr.

We previously reported that Ru65 (Fig. 1a) generates ^1^O_2_ upon UV-A irradiation (350 nm, 2.58 J cm^–2^) and that this results in the relaxation of supercoiled plasmid DNA.^21^ To corroborate these findings and clarify the molecular mechanism of DNA damage, we examined the binding of Ru65 to the plasmid pUC18 (Fig. S1†). The incubation of pUC18 with Ru65 in the absence of UV-A irradiation caused retardation in the mobility of both the supercoiled and nicked forms of the plasmid (Fig. 1b and S1†), confirming the established intercalating properties of Ru65 in DNA.^25^ The incubation of pUC18 with Ru65 followed by UV-A irradiation (350 nm, 0.65–2.58 J cm^–2^) showed a dose-dependent ability of the metal complex to nick plasmid DNA (Fig. 1b).

We reasoned that the generation of ROS, such as ^1^O_2_, in close proximity to DNA may cause damage to bases, with 8-oxo-guanine being a highly mutagenic lesion,^26^ as previously demonstrated upon the light irradiation of other DNA intercalating agents.^27^ LC-MS analysis of guanosine incubated with Ru65 and activated by UV-A irradiation (350 nm, 2.58 J cm^–2^) showed the appearance of peaks that were absent in non-irradiated controls and that corresponded to the sub-products of guanosine oxidation (Fig. 1c). The incubation of pUC18 with Ru65 followed by UV-A irradiation and treatment with formamido-pyrimidine DNA glycosylase (Fpg or 8-oxoguanine DNA glycosylase), an enzyme that releases damaged guanines from dsDNA leaving a one-base gap, showed that supercoiled pUC18 was almost fully converted in the nicked form under these conditions (Fig. 1d).

Collectively, these data indicate that the irradiation of Ru65 intercalated in DNA causes oxidative damage.

### Photo-irradiation of Ru65 blocks cell cycle progression

To elucidate the molecular mechanism of action of Ru65 and to assess the impact of the damage caused to DNA, we performed a set of analyses on living cells. We have previously observed that Ru65 preferentially accumulates into the nucleus of HeLa cells.^21^ To extend this finding, we used confocal microscopy and followed the uptake of Ru65 in a number of cell lines that were grown for 4 h in the presence of the metal complex. Under these conditions, Ru65 displayed nuclear localization in U2OS, MCF7, and CAL33 cancer cells as well as in the normal retinal epithelial cell line RPE-1 (Fig. S2a†). Time-course experiments conducted in the continuous presence of the metal complex revealed an increasing accumulation of Ru65 into the nucleus of U2OS cells over a 24 h period, with a detectable signal 30 min post-administration of the metal complex and then substantial amounts of Ru65 being internalized at 4 h (Fig. S2b†). The incubation of U2OS cells with Ru65 for 4 h, followed by the removal of unbound metal complex, revealed that Ru65 was still present in the nucleus 24 h upon exchange of the medium (Fig. S2c†). Since the luminescence of a target compound detected by confocal microscopy can be quenched in a water environment (light-switch effect), to confirm the data obtained by microscopy, we tracked Ru65 uptake using inductively coupled plasma mass spectrometry (ICP-MS).^28^ The treatment of U2OS cells with Ru65 (50 μM, 4 h) displayed internalization of >60% of the complex present in the medium. Sub-cellular bio-distribution studies showed an accumulation of Ru65 in the nucleus (55%), with ∼35% metal complex present in the residual fraction, which was mainly composed of cytoplasm (Table 1).

**Table 1:** Uptake of Ru65 (50 μM, 4 h) in different compartments of U2OS cells, as determined by ICP-MS; nd = not detected

Ru65	Mitochondria	Nucleus	ER	Residual	Total
Dark	0.18 ± 0.08	0.98 ± 0.15	nd	0.60 ± 25.8	1.76 ± 0.30
UV-A	0.23 ± 0.03	1.52 ± 0.56	nd	0.28 ± 0.15	2.03 ± 0.35

**Fig. 2:**
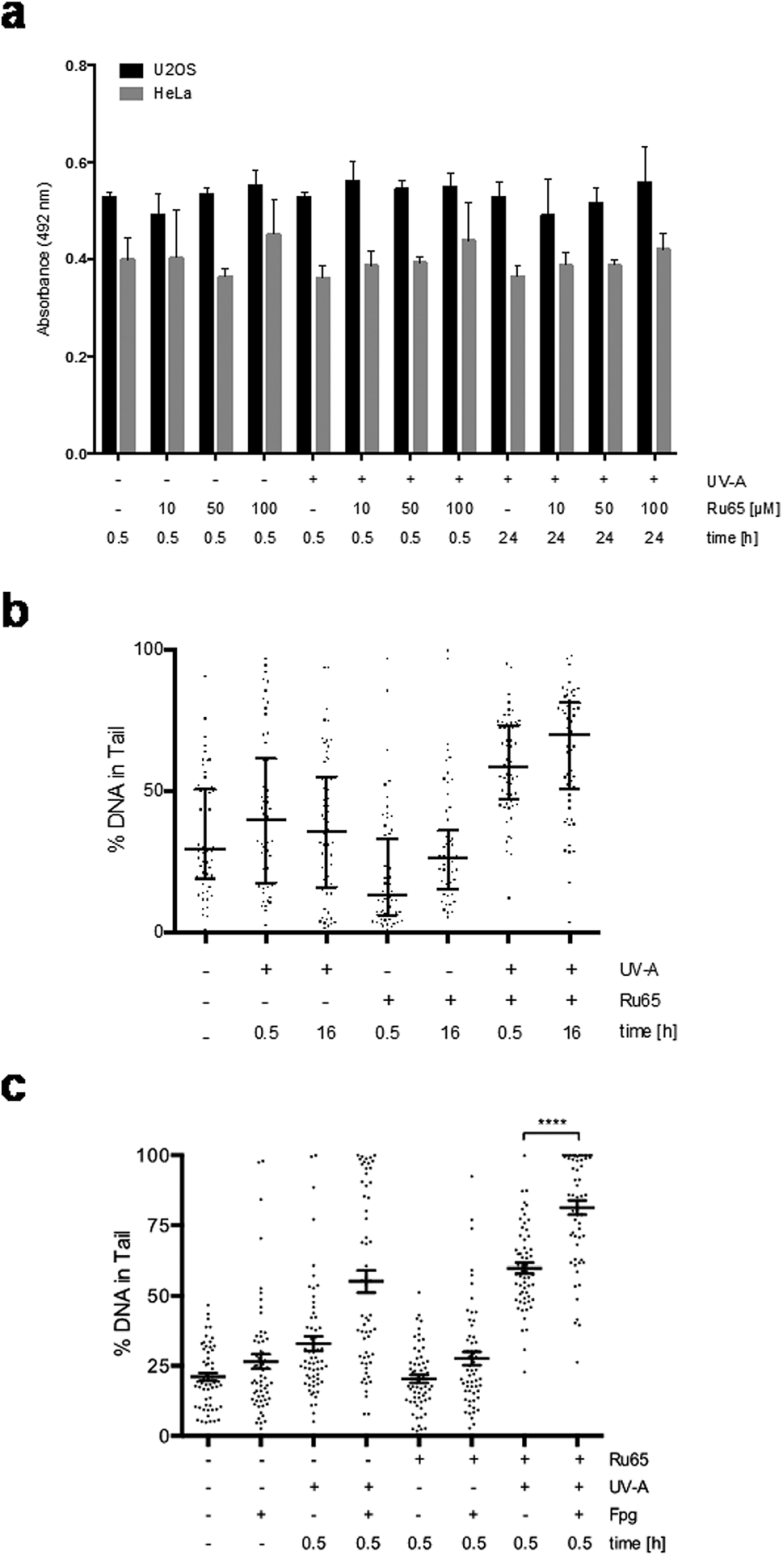
Photo-irradiation of Ru65 causes damage to genomic DNA. (a) Quantification of cyclobutane pyrimidine dimer (CPD) formation with the TDM-2 antibody upon the treatment of U2OS or HeLa cells with Ru65 and UV-A. Mean values of quadruplicate determinations with the standard error of the mean (SEM) are plotted. (b) DNA double- and single-strand breaks in U2OS cells treated with Ru65 and UV-A irradiation were assessed by alkaline comet assay. Each dot represents a single cell. Values are presented as median ± interquartile range. *n* = 60 comets per group. (c) Quantification of oxidative damage to DNA. Agarose-embedded U2OS cells were incubated with Fpg (0.8 U; 1 h at 37 °C) and resolved on alkaline gels. The percentage of DNA in the tail was quantified as described in (b). Data of UV-A irradiated Ru65 were compared using the Mann–Whitney *U* test: ****p < 0.0001.

Experiments conducted on HeLa and U2OS cells ruled out that the UV-A irradiation of Ru65 could cause the formation of cyclobutane pyrimidine dimers (CPDs) (Fig. 2a) or pyrimidine (6-4) pyrimidone photoproducts (6-4PPs) (data not shown). To assess the nature of the DNA damage caused by Ru65 upon light irradiation we used alkaline Single Cell Gel Electrophoresis (SCGE) or comet assay^29^,^30^ and pulse-field gel electrophoresis (PFGE).^31^ Quantification of the amount of DNA present in the comet's tail showed an increase of single- and double-strand breaks at the 0.5 h and 16 h time points over the controls (Fig. 2b and S3†). PFGE confirmed that DNA double-strand breaks and DNA fragmentation occurred in response to the UV-A-irradiation of Ru65 (Fig. S4a†), whereas the administration of UV-A alone at the dose used in this study, and significantly below the dose administered by others working with similar metal complexes,^32^ did not result in significant damage to DNA (Fig. S4b†).

Next, we assessed whether Ru65 would cause base oxidation in living cells similar to that observed on isolated plasmid DNA. Alkaline comet assays confirmed that the metal complex caused damage to DNA upon light irradiation and that the treatment of agarose-embedded cells with Fpg increased the amount of signal in the tail (Fig. 2c).

**Fig. 3:**
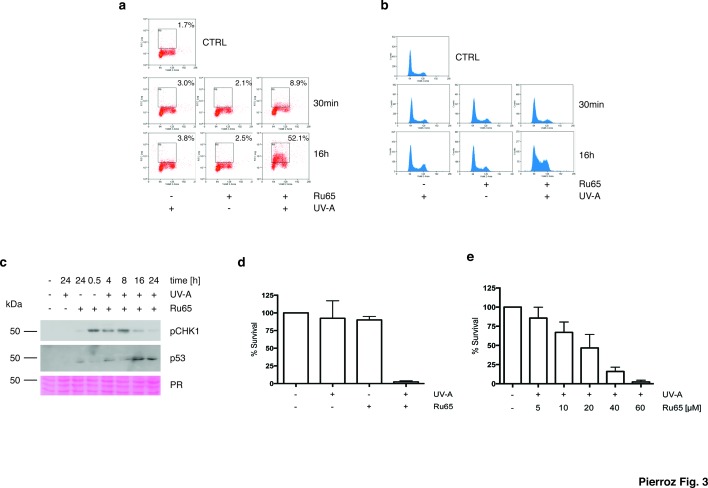
Early and delayed cellular response to the photo-irradiation of Ru65. (a) U2OS cells were treated with Ru65 (50 μM) and UV-A (1.29 J cm^–2^) as indicated. Upon fixation, the cells were probed with an antibody to γH2AX and examined by flow cytometry. (b) U2OS cells treated as described above and stained with DAPI were examined by flow cytometry. (c) Western blot analysis of CHK1 phosphorylation (pCHK1) and p53 expression in U2OS cells treated with Ru65. PR: Ponceau Red. (d) U2OS cells were treated with Ru65 (50 μM) and UV-A (1.29 J cm^–2^) prior to trypsinization and re-seeding at low density. Plotted values are the average of triplicates from three independent experiments with indication of SEM. (e) U2OS cells were treated with increasing amounts of Ru65 and examined as described in (d).

To gain mechanistic insights into the cellular response caused by the light irradiation of Ru65, we examined the presence of γH2AX, an established marker of the DNA damage response (DDR). To this end, we used a flow cytometry-based method that couples the quantification of DNA damage (γH2AX) with analysis of the DNA content (DAPI), according to an established protocol.^33^ Control experiments, where U2OS cells were treated with UV-A alone (1.29 J cm^–2^), showed the transient phosphorylation of H2AX (Fig. S4c†). UV-A irradiation of cells treated with Ru65 showed that the modest H2AX phosphorylation occurring at the early time points was followed by a large increase of the γH2AX signal at 16 h (Fig. 3a). The cell cycle profile showed a marked accumulation of cells at the S- and G2-phases of the cell cycle at 16 h upon the irradiation of Ru65 (Fig. 3b). Western blot analysis of CHK1 phosphorylation, a DDR marker,^34^ and p53 expression confirmed the transient activation of DDR at the early time points and a sustained block of cell cycle progression from 16 h onward (Fig. 3c). Similar results were obtained using CAL33 cells (Fig. S5†), derived from a squamous carcinoma of the tongue and representative of cancers that can be easily reached with light probes.

To further investigate the outcome of the light irradiation of Ru65, we performed viability assays on a set of cell lines. Loss of viability was observed in all cell lines tested following the photo-irradiation of Ru65 (Table 2).

**Table 2:** Cell lines incubated with Ru65 (4 h followed by wash-off) were either non-irradiated (–) or UV-A irradiated (+) and the IC_50_ (μM) was determined. Cisplatin (CDDP) was used as a comparison

	HeLa		U2OS		CAL33		RPE-1 hTERT	
UV-A	–	+	–	+	–	+	–	+
Ru65	>100	20.0 ± 6.1	>100	30.5 ± 2.9	>100	17.4 ± 5.3	>100	35.4 ± 3.3
CDDP	30.9 ± 3.6	27.4 ± 2.3	26.8 ± 1.9	21.6 ± 0.3	30.2 ± 7.2	25.0 ± 4.2	62.3 ± 9.7	64.1 ± 3.8

To extend these observations, we assessed the long-term cellular response to Ru65 by clonogenic survival assays.^35^ Consistent with the data above, cytotoxicity was observed only upon the UV-A irradiation of Ru65 (Fig. 3d). Dose–response studies confirmed a direct correlation between the decrease of survival and Ru65 doses administered to cells (Fig. 3e).

Taken together, these data show that Ru65 preferentially accumulates into the nucleus and, upon activation by low-dose UV-A, causes cell cycle arrest and loss of viability.

### Photo-irradiation of Ru65 triggers cell death by ER-stress pathways

**Fig. 4:**
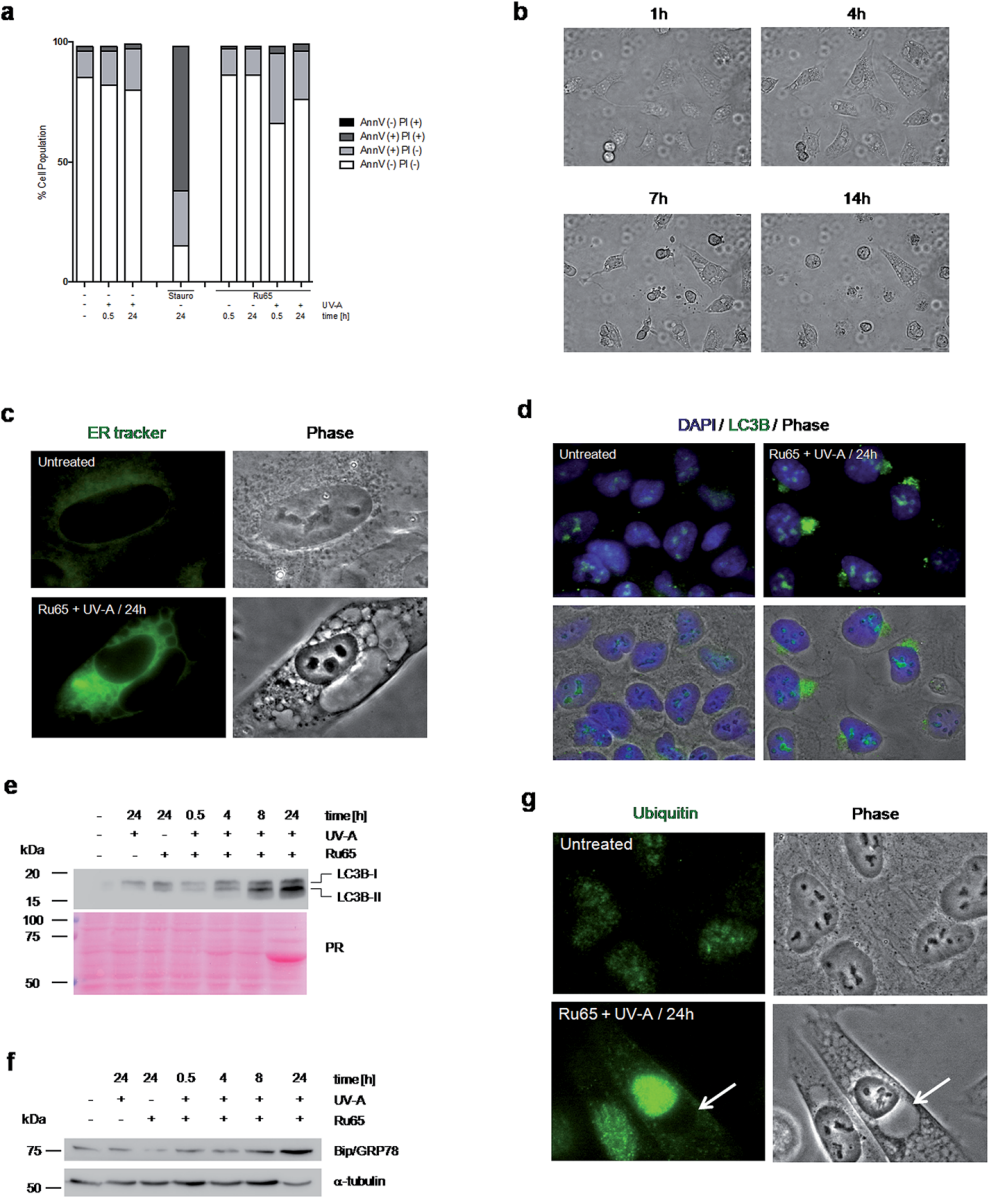
Photo-irradiation of Ru65 triggers ER-stress pathways. (a) U2OS cells treated with Ru65 (50 μM) and UV-A (1.29 J cm^–2^) were fixed and examined for Annexin-V staining and PI uptake. AnnV (–) PI (+): necrotic cells; AnnV (+) PI (+): late apoptotic cells: AnnV (+) PI (–): early apoptotic cells; Ann (–) PI (–): healthy cells. (b) Phase contrast stills of U2OS cells examined at the times indicated upon the photo-irradiation of Ru65. (c) Endoplasmic reticulum (ER) fluorescence staining and phase contrast stills of U2OS cells left untreated or 24 h following the photo-irradiation of Ru65. (d) Immunofluorescence visualization of LC3B expression in U2OS cells treated as in (c). Nuclei were stained with DAPI. (e) Western blot analysis of LC3B expression in U2OS cells treated as in (c). (f) Western blot analysis of BIP/GRP78 expression in U2OS cells treated as in (c). α-Tubulin was used as a loading control. (g) Immunofluorescence visualization of protein ubiquitylation in U2OS cells treated as in (c). Arrows indicate the vacuoles.

To explore the mechanism triggering cell death upon the light irradiation of Ru65, we complemented the cell cycle studies with the analysis of phosphatidylserine translocation through Annexin-V staining at the cell surface, an assay detecting apoptosis, and propidium iodide (PI) uptake, which is indicative of the early plasma membrane collapse characterizing primary necrosis.^36^ The data showed that the cell cycle arrest observed upon the photo-irradiation of Ru65 was not paralleled by a significantly increased Annexin-V level or PI uptake (Fig. 4a), thereby ruling out death by classic apoptotic or necrotic pathways. Visual inspection of the cells undergoing treatment with Ru65 confirmed the absence of apoptotic bodies in the nucleus and rather revealed the time-course formation of vacuoles of increasing size in the cytoplasm (Fig. 4b and ESI Movie S1†). To assess whether the vacuoles appearing in response to Ru65 originate from the endoplasmic reticulum (ER),^37^ we used ER-Tracker Green, a cell-permeant dye that stains the ER in living cells. Fluorescence microscopy showed decoration of the vacuoles border in cells treated with Ru65, confirming the ER origin of the cytoplasmic vacuoles (Fig. 4c and S6†).

Since it was previously reported that cytoplasmic vacuolation-mediated cell death is preceded by an increased expression of microtubule-associated protein light chain 3 (LC3B), an established marker of autophagy, and of Bip/GRP78, an ER-stress marker,^37^ we decided to assess whether this occurred under our experimental conditions. Immunofluorescence (Fig. 4d and S7a†) and Western blot (Fig. 4e) analyses confirmed that the UV-A irradiation of Ru65 led to an increased expression of LC3B-II, which formed dense granules in the cytoplasm. Under these conditions, we also observed an increased expression of Bip/GRP78 (Fig. 4f and S7b†). Mass spectrometric analysis of the cellular proteins at 24 h following the treatment with Ru65 revealed a robust expression of heat-shock proteins (Hsp60) and translation initiation factors (eIF2A) (Fig. S8 and Table S1†), which are established markers of the unfolded-protein stress response (UPR).^38^ Additionally, we observed that the extent of nuclear protein ubiquitylation was increased in cells treated with Ru65 (Fig. 4g and S9†).

Taken together, these data show that Ru65-dependent cell death is the result of damage occurring in the nucleus and involves ER-mediated stress response pathways.

### Photo-irradiation of Ru65 hampers the execution of mitosis

**Fig. 5:**
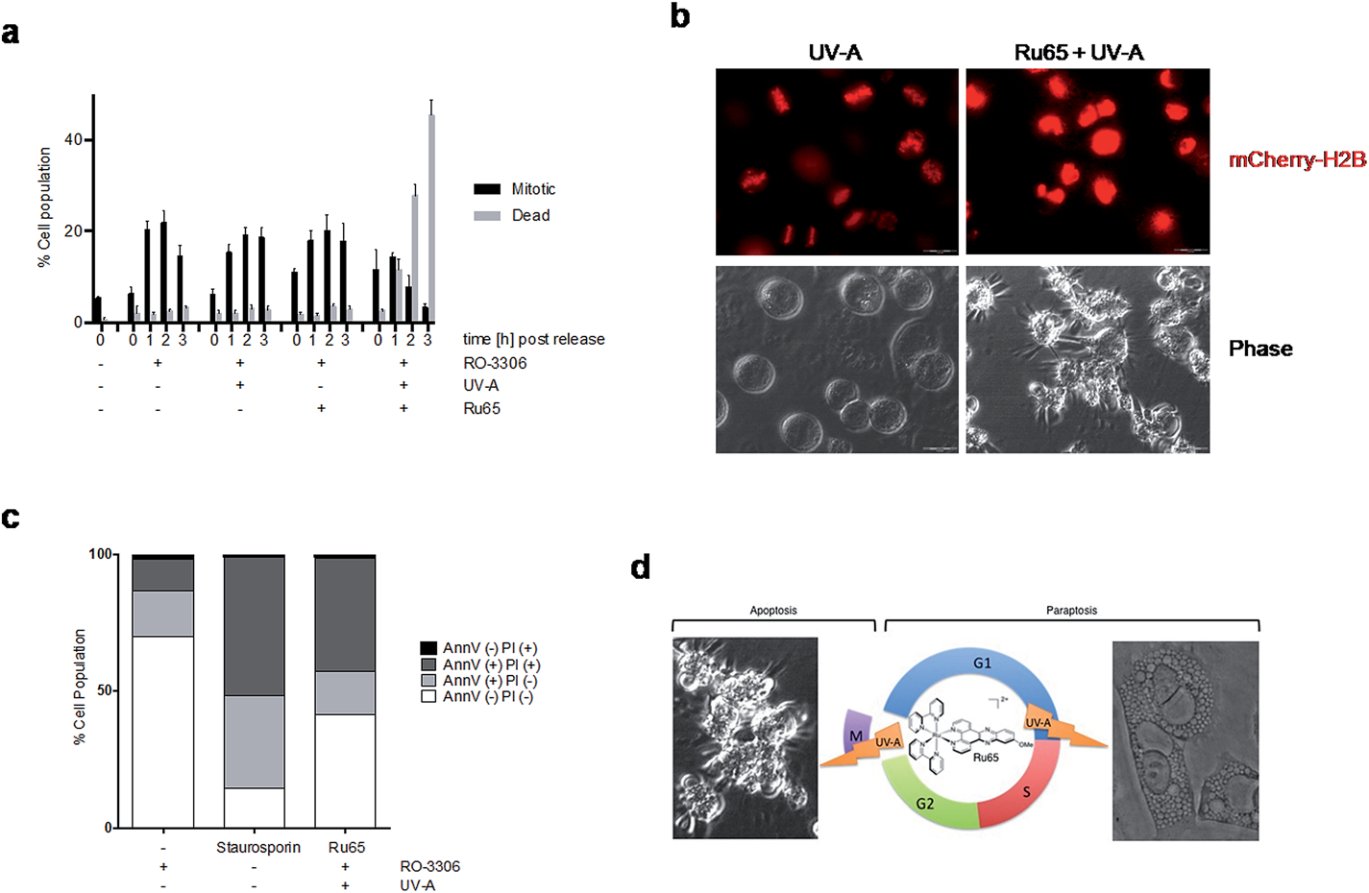
Photo-irradiation of Ru65 hampers the execution of mitosis. (a) HeLa cells were synchronized with RO-3306 (9 μM, 15 h) and treated with Ru65 (50 μM) for the last 2 h of incubation. Cells were irradiated with UV-A (1.29 J cm^–2^) at the time of release from RO-3306. Mitotic and dead cells were quantified at the indicated time points (*n* < 1000 cells). (b) Phase contrast and fluorescence stills of HeLa mCherry-H2B cells treated as in (a). (c) HeLa cells treated as in (a) were fixed and examined for Annexin-V staining and PI uptake. (d) Scheme of the different cell death modes triggered by the UV-A irradiation of Ru65 in interphase or at mitosis.

In the course of our studies, we observed that the photo-irradiation of Ru65 in cells transiting through mitosis severely affected viability. To precisely assess the nature of the cell cycle arrest observed upon the photo-irradiation of Ru65, we performed studies on cells synchronized at the G2/M transition of the cell cycle with the selective and reversible CDK1 inhibitor RO-3306.^39^ In this set of experiments, Ru65 was added during the last 2 h of synchronization. Upon release from RO-3306, the control cells showed a timely progression through mitosis (Fig. 5a and b, S10, and Movie S2†) and transition to the next G1 (Fig. S11†). On the other hand, cells in which Ru65 was photo-irradiated at the time of release from RO-3306 could not complete mitotic transition and died before reaching G1 (Fig. 5a and b, S10, S11, and Movie S3†). Annexin-V and PI staining showed that the photo-irradiation of Ru65 at this point of the cells cycle caused death through apoptotic pathways (Fig. 5c). Cell viability assays conducted on RO-3306 synchronized cells in which Ru65 was added during the last 2 h of synchronization and that were then photo-irradiated at the time of release from the G2/M arrest showed a 3.6-fold reduction of the IC_50_ compared to non-synchronized cells (Table 3).

**Table 3:** HeLa cells were synchronized with RO-3306 (9 μM, 15 h) and Ru65 was added for the last 2 h of incubation. Upon release from the G2/M arrest and wash-off of unbound Ru65, cells were non-irradiated (–) or UV-A irradiated (+) and the IC_50_ (μM) was determined

	HeLa			
UV-A	–	–	+	+
RO-3306	–	+	–	+
Ru65	>100	>100	38.0 ± 6.2	10.4 ± 3.4

On the other hand, the photo-irradiation of Ru65 at 40 min following release from the RO-3306 arrest point, namely when cells synchronously execute mitosis,^39^ did not substantially affect the completion of mitosis (Fig. S12†).

These data indicate that the photo-irradiation of Ru65 causes the rapid death of mitotic cells through pathways that are distinct from those involved in the death of interphase cells.

## Discussion

The application of Ru(ii) polypyridyl complexes in cell imaging and in PDT is well documented.^16^,^20^,^40^–^42^ With regard to their molecular mode of action, the ability of Ru complexes to bind canonical and non-canonical DNA structures, among others, has been reviewed at length.^16^ We have recently reported the synthesis, characterization, photophysical properties, and biological evaluation of a set of substitutionally inert polypyridyl Ru(ii) complexes. Notably, we demonstrated the ability of such complexes to produce ^1^O_2_ in response to light irradiation at a defined wavelength and proposed their application as PSs in PDT.^21^ The recent announcement that a Ru(ii) complex will soon enter clinical trial as a PDT agent against bladder cancer further emphasizes the interest in such compounds.^20^

In the study reported herein, we examined the molecular mechanism of action of one such complex (Ru65) upon light irradiation (Fig. 1a). We observed that Ru65 is efficiently internalized in a number of cell lines, where it mainly localizes in the nucleus (Fig. S2†) and exerts specific cytotoxicity upon irradiation with UV-A (Table 1). Targeting PSs to the nuclear compartment, a site that is particularly sensitive to active oxygen species-induced damage, has been previously attempted with Chlorin e_6_.^22^ However, whereas in the case of Chlorin e_6_ the linking of a nuclear localization signal (NLS) was essential to deliver the molecule to the nucleus, thus bypassing its cytotoxic side-effects on the plasma membrane, Ru65 naturally accumulates in the nucleus. Furthermore, the dose of UV-A irradiation used in our experiments to trigger reactive oxygen species by Ru65 (Fig. S4†) is ∼4-fold lower than that reported by Gicquel *et al.*^32^ and is comparable to that locally employed in combination with psoralen for the treatment of psoriasis, which is generally innocuous for skin types >II.^43^

Using a set of biochemical and biological assays, we demonstrated that Ru65 causes guanine oxidation on isolated plasmid DNA upon light irradiation (Fig. 1) as well as in living cells (Fig. 2). Among purines, guanine is characterized by a low redox potential, rendering it prone to oxidation.^44^ 8-Oxo-7,8-dihydroguanine (8-oxo-G) is one of the most stable and miscoding lesions caused by ROS^26^ and is normally addressed by the Base Excision-Repair (BER) pathway.^45^ The transient nature of the DNA damage response that we observed at the early time points upon the UV-A irradiation of Ru65, monitored through the pattern of CHK1 phosphorylation, likely reflected the repair of DNA base oxidation and single-strand breaks (Fig. 2 and 3). Using structurally similar DNA intercalating Ru(ii) polypyridyl complexes, Gicquel and colleagues argued that their complexes must cause single- and double-strand breaks, based on the ability of specific DNA repair proteins to bind damaged DNA.^32^ In our study, using comet assays as well as pulse-field gel electrophoresis (Fig. 2 and S3†) and a much lower amount of UV-A light doses, we provided formal and direct demonstration that DNA double-strand breaks and DNA fragmentation (Fig. S4†) occur at the time of cell cycle arrest (Fig. 3). Since cell death ensues (Fig. 4), we concluded that the pronounced H2AX phosphorylation and severe DNA damage that parallel the cell cycle arrest upon the photo-irradiation of Ru65 (Fig. 3 and S3–S5†) are the likely consequences of triggered death programs rather than part of a productive DDR. Furthermore, the concomitant ubiquitylation of nuclear proteins and the ongoing unfolded-protein stress response (UPR), encompassing an increased level of translation factors and molecular chaperones and resulting in the formation of ER-derived cytoplasmic vacuoles^46^ (Fig. 4 and S6–S9, Table S1, and Movie S1†) indicate that DNA is likely not the only target of Ru65 in the nucleus upon light irradiation.

In proliferating cells, a prolonged arrest before mitosis appeared to precede the loss of viability and ultimately cell death in response to the photo-irradiation of Ru65 (Fig. 3 and 4). We observed that mitotic cells were much more sensitive to the photo-irradiation of Ru65 than cells in other phases of the cell cycle. Hence, we investigated the effect of the photo-irradiation of Ru65 in cells synchronized at the G2/M transition of the cell cycle using the reversible inhibition of CDK1. The photo-irradiation of Ru65 upon release from the G2/M arrest point hampered the execution of mitosis (Fig. S10 and S11†) and led to massive and rapid death through classic apoptotic pathways (Fig. 5 and S10†). Under these conditions, the IC_50_ for the light irradiation of Ru65 was reduced by 3.6-fold compared to the value observed for non-synchronized cells (Table 3). On the other hand, the photo-irradiation of Ru65 at times when the bulk of synchronized cells transit through mitosis,^39^ did not effectively stop this process (Fig. S12†). This indicates that either Ru65 intercalated into condensed DNA is less suitable to photo-irradiation and therefore less capable of inducing oxidative damage or that Ru65 targets component of the machinery driving entry into mitosis but it becomes ineffective when the latter has been initiated. Future studies will address these issues.

As a whole, our study reached two important conclusions. The first is the elucidation of the mechanism of action of a Ru(ii) polypyridyl complex upon light irradiation. The irradiation of Ru65 in cycling cells using innocuous UV-A light leads to cell cycle arrest, a loss of viability, and death through ER-mediated stress response pathways. DNA breaks and DNA fragmentation detected at the point of cell cycle arrest following the generation of reactive oxygen species by the irradiation of Ru65 are the likely consequences of activated cell death programs and not genuine attempts to repair DNA, a choice that would seriously compromise genome stability. Such a mode of action of photo-irradiated Ru(ii) polypyridyl complexes represents an advantage over classic anticancer chemotherapeutics, which often cause secondary malignancies due to their ability to permanently modify DNA in cells surviving the treatment.^1^ The second conclusion is that the photo-irradiation of Ru65 in mitotic cells results in a rapid induction of cell death at a concentration 3.6-fold lower with respect to the dose causing a loss of viability in non-synchronized cells, paving the way for the implementation of novel therapeutic protocols, according to which cancer patients could be treated with a combination of cell cycle inhibitors and Ru65/light for an effective clearance of tumors.

## Experimental procedures

### Photo-irradiation settings

UV-A treatment was performed in a Rayonet RPR-200 photochemical reactor (Rayonet Corp., Branford, CT, USA) containing six bulbs (14 W each) emitting in the 300–400 nm range (350 nm maximum intensity). The light intensity (55 W m^–2^) was determined using an X11 optometer (Gigahertz-Optik, Germany).

### Immunofluorescence staining and analysis

The cellular localization of fluorescent ruthenium complex was assessed by fluorescence microscopy. Cells were grown on 18 mm Menzel-Gläser coverslips (Menzel-Gläser, Germany) at a density of 2.5 × 10^5^ cells per ml and incubated for 4 h with 100 μM Ru65 at 37 °C. Cells were fixed in 4% formaldehyde and mounted on the slides for viewing by confocal microscopy on a CLSM Leica SP5 microscope (Leica Germany). Ru65 was visualized using the red wavelength selection (ex, 458 nm; em, 600–650 nm) on the CLSM Leica SP5 microscope.

For LC3 and ubiquitin staining, U2OS cells were seeded at a density of 3 × 10^5^ cells per ml in ibiTreat dishes (Ibidi, Martinsried, Germany). After 24 h, the cells were treated for 4 h with Ru65 (50 μM), the medium was replaced, cells were UV-A irradiated (1.29 J cm^–2^), and then placed back in to the incubator for 24 h. Cells were fixed for 15 min at room temperature (RT) in 4% formaldehyde, permeabilized in 0.1% Triton X-100 for 5 min at 4 °C, blocked in 3% milk/PBS, and then incubated overnight at 4 °C with anti-LC3-B or anti-ubiquitin antibodies. After washing in 3% milk/PBS, AlexaFluor 488 goat-anti mouse/rabbit antibodies (1 : 1000) were added for 1 h at 37 °C, followed by 15 min staining in DAPI solution (1 μg ml^–1^). Cells were washed and overlaid with PBS for viewing by microscopy on an Olympus IX 81 motorized inverted microscope (Olympus, Hamburg, Germany).

To visualize the ER, U2OS cells were seeded and grown as above. After 24 h, the cells were treated for 4 h with Ru65 (50 μM) and with ER-Tracker Green (BODIPY FL Glibenclamide) (1 μM) for the last 1.5 h, the medium was then replaced and the cells were UV-A irradiated (1.29 J cm^–2^). Cells were fixed in 4% formaldehyde, stained for 15 min with DAPI solution (1 μg ml^–1^), washed, and overlaid with PBS for viewing as indicated above.

### Pulse-field gel electrophoresis

Sub-confluent cultures of U2OS were treated with vehicle alone (DMSO), camptothecin (CPT 1 μM), or Ru65 (50 μM) and were either non-irradiated or UV-A irradiated. Cells were harvested by trypsinization, and agarose plugs of 10^6^ cells were prepared in a disposable plug mold (Bio-Rad). Plugs were incubated in lysis buffer (100 mM EDTA, 1% (w/v) sodium lauryl sarcosyl, 0.2% (w/v) sodium deoxycholate, 1 mg ml^–1^ proteinase K) at 37 °C for 72 h, and washed four times in 20 mM Tris–HCl pH 8.0, 50 mM EDTA before loading onto an agarose gel. Electrophoresis was performed for 23 h at 14 °C in 0.9% (w/v) Pulse Field Certified Agarose (Bio-Rad) containing Tris-borate/EDTA buffer according to the conditions described in^47^ and adapted to the Bio-Rad CHEF DR III apparatus. The gel was finally stained with ethidium bromide (EtBr) and analyzed using an Alpha Innotech Imaging system.

## Conflict of interest

The Authors declare no competing financial interests in relation to the work described here.

## Abbreviations

bipy: 2,2′-Bipyridine
dppz: Dipyrido[3,2-*a*:2′,3′-*c*]phenazine
ATM: Ataxia-telangiectasia mutated kinase
ATR: Ataxia-telangiectasia and RAD3-related kinase
CHK1: Checkpoint kinase 1
ER: Endoplasmic reticulum
H2AX: Histone 2AX
ICP-MS: Inductively coupled plasma mass spectrometry

## Supplementary Material

Supplementary information

Supplementary movie

Supplementary movie

Supplementary movie
