# Supplementary material for: Dual mode of cell death upon the photo-irradiation of a RuII polypyridyl complex in interphase or mitosis
Source: Chem Sci. 2016 Jun 1;7(9):6115–24. doi: 10.1039/c6sc00387g (PMC5032677; doi:10.1039/c6sc00387g)
Supplement: Supplementary file 1 [file SC-007-C6SC00387G-s001.pdf]

Electronic Supplementary Material (ESI) for Chemical Science.
This journal is © The Royal Society of Chemistry 2016

Supplementary Information

Dual mode of cell death upon photo-irradiation of a Ru^{II} polypyridyl complex in interphase or mitosis

Vanessa Pierroz,[1,2] Riccardo Rubbiani,[2] Christian Gentili,[1] Malay Patra,[2] Cristina Mari,[2] Gilles Gasser[2,3] and Stefano Ferrari[1,3]

[1]Institute of Molecular Cancer Research, University of Zurich, Winterthurerstrasse 190, CH-8057 Zurich, Switzerland

[2]Department of Chemistry, University of Zurich, Winterthurerstrasse 190, CH-8057 Zurich, Switzerland

Supplementary Methods

Antibodies, chemicals and enzymes – Rabbit monoclonal antibodies to phosphorylated histone H2AX (#7631) and phosphorylated CHK1 (#2348) as well as rabbit polyclonal antibody to BiP/GRP78 (#3183) were purchased from Cell Signaling Technology (Beverly, MA, USA). Mouse monoclonal antibody to Ubiquitin (P4D1, sc-8017) and rat monoclonal antibody to α-tubulin (YOL 1/34, sc-53030) were from Santa Cruz Biotechnology (Santa Cruz, CA, USA). Rabbit polyclonal antibody to LC3B (L7543) was from Sigma-Aldrich (Buchs, Switzerland). Mouse monoclonal antibody to p53 (clone PAb421, SA293, was from Biomol (Hamburg, Germany). The TDM-2 and 64M-2 antibodies were purchased from Cosmo Bio (Japan). HRP-conjugated anti-rat secondary antibody was from Santa Cruz Biotechnology. HRP-conjugated anti-mouse and anti-rabbit secondary antibodies were obtained from Amersham-Biosciences/GE-Healthcare (Otelfingen, Switzerland). The Alexa Fluor® 488 anti-rabbit secondary antibody was from Cell Signaling Technology. Formamido-pyrimidine DNA Glycosylase (Fpg) was purchased at New England Biolabs (Ipswich, MA, USA). The CometAssay® kit was from Trevigen (Gaithersburg, MD, USA). Crystal violet was obtained from Sigma-Aldrich (Buchs, Switzerland). The selective CDK1 inhibitor RO-3306 (Roche, Switzerland)[1] was dissolved in DMSO at 9 mM stock concentration and stored in aliquots at -20 °C.

Cell culture - U2OS cells and hTERT-immortalized retinal pigment epithelial cells RPE-1 were cultured in DMEM (Gibco, Life Technologies, USA) supplemented with 10% fetal calf serum (FCS) (Gibco). HeLa and Hela mCherry H2B cells were maintained in DMEM containing 5% FCS. MCF-7 cells were grown in MEM (Gibco) supplemented with 10% FCS and 200 mM L-Glutamine. CAL-33 cells were cultured in RPMI (Gibco) without phenol red supplemented with 10% FCS and 1% Glutamax (Gibco). All cell lines were complemented with penicillin (100 U/mL), and streptomycin (100 µg/mL) and stored in a humidified incubator containing 5% CO_2 at 37 °C.

Guanine Light-mediated Oxidation - Guanine (Gua) was dissolved in 1 M aqueous HCl and underwent several gently heating steps to yield a 200 mM clear stock solution. An aliquot of the stock solution was further diluted in acetonitrile (0.5:10 v/v) and treated with Ru65 (final concentration 200 µM). Due to singlet oxygen quenching effects of hydrophilic solutions, to optimize the $^1O_2$ production *in situ*, acetonitrile was chosen as medium. The sample was irradiated at 350 nm for 10 min (2.58 J/cm^2) at room temperature in a Rayomet Chamber Reactor and, after a further 1:1 (v/v) dilution in acetonitrile, analyzed *via* mass spectrometry. The sub-products of

guanine oxidation, namely deoxyguanidinohydantoin (dGh) and (Gh) guanidinohydantoin,[2] were detected upon injection in an Esquire 6000 ESI mass spectrometer (Bruker). Spectra were recorded in positive mode. A series of controls of the mixture, Ru65 alone or guanine alone kept in the dark were performed. Phenalenone (Phen) was used as positive control at a concentration of 100 µM. The following mass (m/z, in g/mol) were detected: Gua $C_5H_5N_5O$ expected [M] 151.1, found $[M+H]^+$ 152.0, [Gua-dimer+Na] 325.1; Ru65 $C_{39}H_{28}F_{12}N_8OP_2Ru$ expected $[M]^+$ 726.1, found $[M]^{2+}$ 363.1; dGh $C_4H_5N_5O_2$ expected $[M]^+$ 155.1, found $[M\text{-dimer}+H]^+$ 311.0; Gh $C_4H_7N_5O_2$ expected $[M]^+$ 157.1, found $[M]^+$ 156.9; Phen $C_{13}H_8O$ expected [M]+ 180.2, found $[M+H]^+$ 181.0[2].

Alkaline comet assay – Alkaline comet assays were performed using the CometAssay Reagent Kit (Trevigen), according to the manufacturer's instructions. Briefly, U2OS cells were seeded at a concentration of 3 x 10^5 cells/ml in a 6 cm dish one day before treatment. The next day cells were treated and irradiated as indicated above and let recover for 30 min or 16h. After resuspension in PBS at a concentration of 1 x 10^5 cells/ml and combined with LMAgarose at a ratio of 1:10 (v/v), 50 µl of the mixture was loaded onto CometSlide and placed at 4 °C for 30min. Embedded cells were lysed in a bath at 4 °C for 30min, slides were immersed in alkaline unwinding solution for 20 min at RT prior to alkaline electrophoresis (21 V, 30min, in alkaline electrophoresis solution). Following two baths in H_2O of 5min and one of 70% EtOH of 5 min, samples were dried at 37 °C and stained with SYBR green for 30min. A total of 60 cells were scored using an epifluorescence microscope (Leica) with 20x magnification objective and Comet Assay IV analysis system (Perceptive Instruments, UK) to quantify DNA in the tail. Statistical analysis was performed using GraphPad Prism software (GraphPad, San Diego, CA, USA). Results are presented as mean values for % of DNA in tail.

Flow Cytometry - Cells were seeded at a concentration of 3 x 10^5 cells/ml in a 6 cm dish one day before treatment. The next day cells were treated with Ru65 and UV-A irradiated (1.29 J/cm^2). For control UV-C treatment, cells were set in a Stratalinker 1800 (Stratagene, Agilent Technologies, USA) and irradiated with 20 J/m^2. Cells were fixed in 4% formaldehyde for 15min at room temperature (RT) and permeabilized in 0.1% Triton-X100/PBS for 10min on ice. Upon washing with 1% BSA/PBS cells were resuspended in 1% saponin dissolved in 1% BSA/PBS buffer containing the primary antibody (rabbit anti γH2AX 1:250) for 2h at RT. Cells were washed with saponin/BSA/PBS and the secondary antibody (anti-rabbit or anti-mouse Alexa Fluor 488 1:50) was added in the same buffer for 30min at RT in the dark. Cells were washed with saponin/BSA/PBS followed by 1% BSA/PBS before resuspension in 1% BSA/PBS containing 0.1 mg/ml RNase and

1μg/ml DAPI at RT in the dark for 30min. Alexa Fluor 488 and DAPI signals were acquired on 10'000 cells / sample using a CyAn ADP 9 flow cytometer (Dako) and analyzed with Summit 4.3 software.

Cell viability and colony formation assays - Cytotoxicity of the Ru(II) complex was assessed by a fluorometric cell viability assay using resazurin (Promocell, Germany). Briefly, one day before treatment, cells were seeded at a density of $4x10^3$ cells/well in 100 μl in 96-well plates and the next day incubated for 4h with either Ru65 complex or cisplatin. Upon replacement of the medium and UV-A irradiation (1.29 J/cm^2), cells were returned to incubator for 48h and viability assessed as previously described.[3]

For colony formation assays cells were seeded at 3 x 10^5 cells/ml in a 6 cm dish and the next day treated and irradiated as above. Cells were trypsinized and re-seeded in triplicate in 6 well dishes. Single colonies were stained with a crystal violet/ethanol solution (0.5%/20%) after 12-14 days of culturing and counted. The survival fraction was determined by the ratio of plating efficiency for treated and untreated cells.

Sample preparation for inductively coupled plasma-mass spectrometry (ICP-MS). U2OS cells were seeded a week before treatment at a concentration of $1x10^6$ cells/ml in 175 cm^2 cell culture flask, grown until 80% of confluence and incubated with Ru65 (50 μM, 4h). The medium was replaced and cells were irradiated at 350 nm for 5min in Rayonet Chamber Reactor Comple (1.29 J/cm^2). After 30min recovery, cells were trypsinized and collected by centrifugation (5910R, Eppendorf) at 650 g for 5min at 4 °C. The different cellular organelles were isolated *via* differential centrifugation following established methods.[4] Briefly, cell pellets were re-suspended in 1.5 ml of extraction buffer (10 mM Hepes, 250 mM sucrose, 25 mM KCl, 1 mM EGTA, pH 7.8) supplemented with protease inhibitors cocktail (Cat. Nr. P8340, Sigma Aldrich) and incubated for 10min on ice. Cells were lysed with a pre-chilled Dounce homogenizer (7 ml, tight pestle A, 15-20 strokes) and centrifuged at 600 g for 10min at 4 °C. The pellet was re-dissolved in 1.0 ml of a sucrose solution (0.25 M sucrose, 10 mM $MgCl_2$) and layered with 1.0 ml of a second hypertonic sucrose solution (0.50 M sucrose, 0.5 mM $MgCl_2$). The suspension was centrifuged at 1500 g and 4 °C for 10min. The pellet was re-suspended in 2 ml of the second sucrose solution and centrifuged at 1450 g and 4 °C for 5min to obtain the nuclear fractions. All the steps of the isolation procedure were monitored under phase contrast microscope on Menzel-Gläser coverslips (Olympus IX81 microscope). The supernatant was transferred to a new tube and centrifuged at 11000 g for 15min at 4 °C. The obtained pellets were re-dissolved in a mitochondria extraction buffer (10 mM Tris-HCl

pH 6.7, 0.15 mM $MgCl_2$, 0.25 M sucrose, 1 mM DTT) and further centrifuged at 11000 g for 15min at 4 °C. The final pellets represented pure mitochondrial fractions. Supernatants of the first mitochondrial extraction were transferred to a polycarbonate u-centrifuge tube (Beckman Coulter) and centrifuged at 43000 g for 1h in an Optima Max-XP Ultracentrifuge (Beckman Coulter). The obtained pellet was re-dissolved in 200 µL of extraction buffer (see above) and represented pure ER microsomal extracts. All the supernatant phases discarded during the isolation of nuclei and mitochondria were collected and represented the "residual" fraction. An aliquot of crude lysate, nuclear, mitochondrial (lysed by *freeze and thaw* cycles followed by 20min incubation in ultrasonic bath) and residual fractions was used for protein quantification. An aliquot of the intact ER-microsome fraction was used for evaluation of integrity by monitoring the processing of NADPH at 340 nm and using human liver microsomes (Gibco Thermo Fisher) as positive control. All fractions were isolated from the same cellular sample for direct comparative purposes. The isolated fractions were lyophilized on an Alpha 2-4 LD plus (CHRIST) and chemically digested with 1 mL of a 20% nitrohydrochloric acid solution (HCl:HNO_3, 3:1) for 24h. The resulting suspensions were filtered on 0.20 µm non-pyrogenic sterile Filtropur filters (Sarstedt) and brought to 10 mL with 2% nitrohydrochloric. The obtained samples were injected in ICP-MS.

ICP-MS studies. ICP-MS measurements were performed on an Agilent QQQ 8800 Triple quad ICP-MS spectrometer (Agilent Technologies) with a ASX200 autosampler (Agilent Technologies), equipped with standard nickel cones and a "micro-mist" quartz nebulizer fed with 0.3 ml/min analytic flow (as a 2% HNO_3 aqueous solution). Ruthenium was measured against a Ru single element standard (Merck 170344.100) and verified by two controls (Agilent5188-6524 PA Tuning 2 and a freshly prepared mix of Ru, Ni and Nb 1 ppb standards). Ruthenium content of the samples was determined by means of a 7-step serial dilution in the range between 0 and 300 ppb of Ru ($R>0.99$) with a background equivalent concentration of BEC: 8.5 ppt and a detection limit of DL: 28.4 ppt. The isotope ^{99}Ru (12.76% abundance) ^{101}Ru (17.06% abundance) was evaluated in "no-gas" mode and He-gas mode. Spiking the samples with untreated negative controls (to account for eventual carbon content from the biological samples) resulted in equivalent values within error ranges. A solution of Indium (100 ppb) and Tungsten (100 ppb) was used as internal standard. The results are expressed as nmol Ru / mg protein (correction due to the different mass of the observed cellular compartments), as mean ± StDev of different independent experiments.

Quantification of cyclobutane pyrimidine dimers (CPDs) and pyrimidine (6-4) pyrimidone photoproducts (6-4PPs) – CPDs and 6-4PPs were quantified of by ELISA-based assays. Briefly,

genomic DNA was extracted from cells at given time-points, deposited on a protamine sulfate-precoated 96-well microtiter plate at a concentration of 1ng/well for CPDs and 200 ng/well for 6-4PPs in quadruplicate. CPDs and 6-4PPs were detected with the TDM-2 and 64M-2 antibodies, respectively [5], according to the manufacturer's instructions.

Western blotting – Cell lysis and immunoblot analysis were performed as previously described [6]. Proteins were revealed using the Western blotting detection kit WesternBright™ ECL (Advansta, Menlo Park, CA, USA) and signal imaged using the Fusion Solo system (Vilber Lourmat).

Apoptosis and necrosis assays – Induction of apoptosis or necrosis was determined by the AnnexinV / propidium iodide assay using flow cytometry and according to the manufacturer instructions (BD Pharmingen, #556419).[7, 8] Briefly, cells undergoing treatment Ru65 were collected by trypsinization, centrifuged and resuspended in binding buffer (10 mM Hepes, 140 mM NaCl, and 2.5 mM $CaCl_2$, pH 7.4) at a concentration of 1 x 10^6 cells / ml. Pellets (100 µl) were transferred into a FACS culture tube (1 x 10^5 cells) and 5 µl of Annexin V-FITC complex solution and 5 µl of a 50 µg / ml propidium iodide (PI) solution were added. Samples were incubated for 15min at RT in the dark, 400 µl of binding buffer were added and the probes analyzed with a CynAn ADP9 flow cytometer with the FITC (for Annexin V-FITC, excitation = 488 nm, emission = 515-545 nm) and PE-Texas Red channels (for PI, excitation = 488 nm, emission = 564-606 nm). Data were analyzed with Summit v4.3 software. Staurosporine (0.5 µM, 4h followed by wash-off) as well as untreated cells in the dark or cells irradiated in the absence of Ru65 were used as controls.

Supplementary Table

Table S1 - List of proteins identified in the Coomassie blue band marked with an asterisk in Fig. S8. The number of unique peptides identified for each entry are indicated.

Gene	Protein	Acc Nr	Mw	CTRL	Ru65+UV-A
HSPD1	60 kDa heat shock protein	P10809	61	11	27
HSP90AB1	Heat shock protein HSP 90-beta	P08238	83	2	13
CCT3	T-complex protein 1 subunit gamma	P49368	61	2	12
CCT5	T-complex protein 1 subunit epsilon	P48643	60	0	12
EIF2A	Eukaryotic translation initiation factor 2A	Q9BY44	65	0	9
PPP2R1B	Serine/threonine-protein phosphatase 2A 65 kDa regulatory subunit A alpha isoform	P30154	66	0	6
EIF3L	Eukaryotic translation initiation factor 3 subunit L	Q9Y262	67	0	7
NARS	Asparagine--tRNA ligase	O43776	63	0	5
TCP1	T-complex protein 1 subunit alpha	P17987	60	0	7
CCT7	T-complex protein 1 subunit eta	Q99832	59	0	2

Supplementary Figures

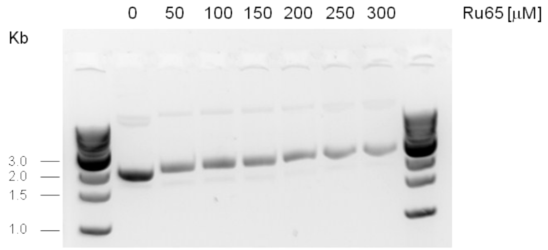

Figure S1 - DNA intercalation by Ru65 (related to Fig. 1)

The plasmid pUC18 (150 ng) was incubated with the indicated amounts of Ru65 for 30min. Products were resolved on a 1% agarose gel in the presence of EtBr.

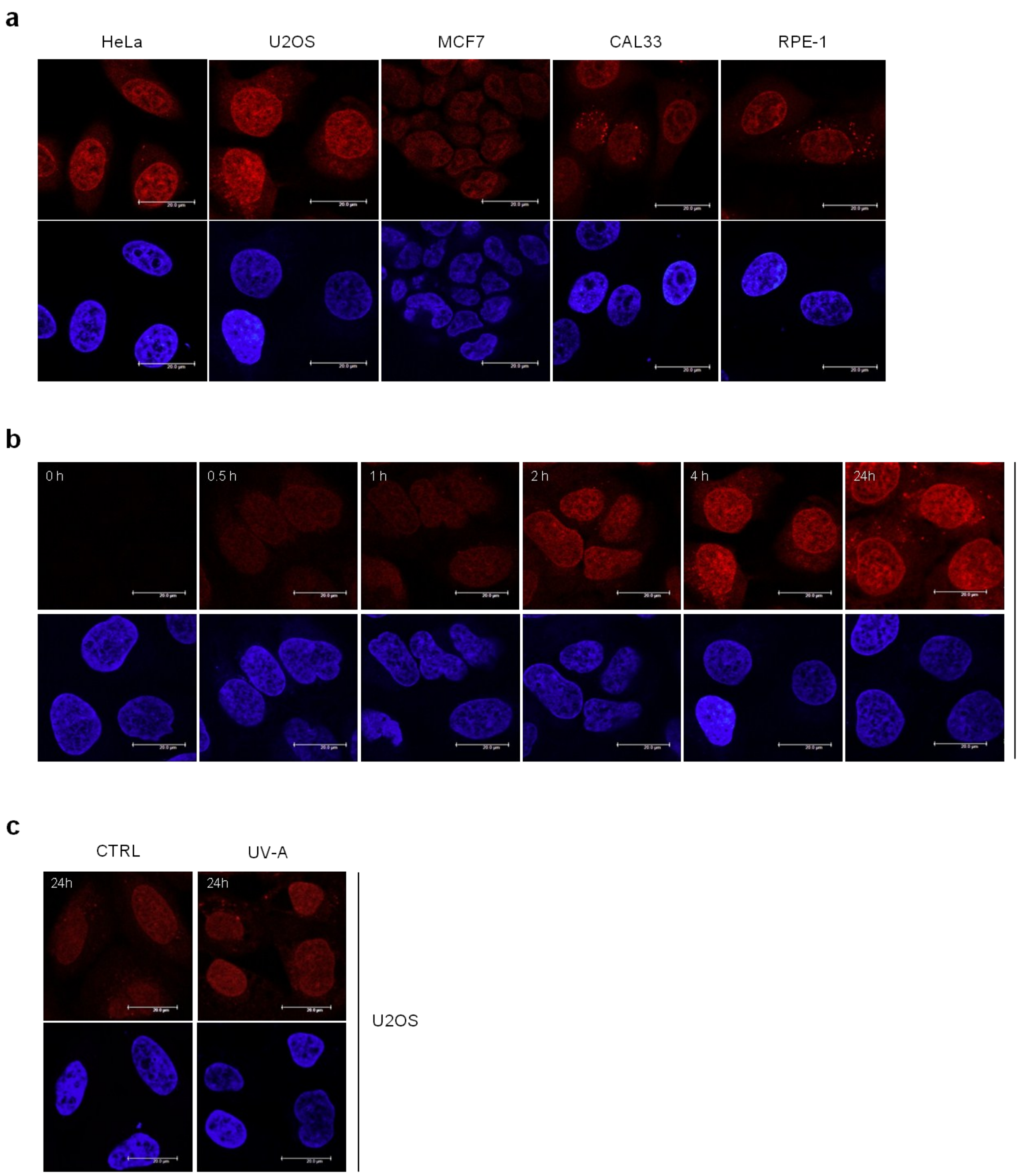

Figure S2 – Ru65 localizes to the cell nucleus

a. Cancer cell lines (HeLa, U2OS, MCF7 or CAL33) and normal RPE-1 cells were treated with Ru65 (100 µM, 4h), the complex was removed and cells were fixed and visualized by confocal microscopy. Scale bar = 20µm.

b. Time course uptake of Ru65 (100µM) in U2OS. Cells were fixed and visualized as described in a.

c. Localization of Ru65 (100μM) in U2OS 24 h upon removal of the complex. Cells were fixed and visualized as described in a.

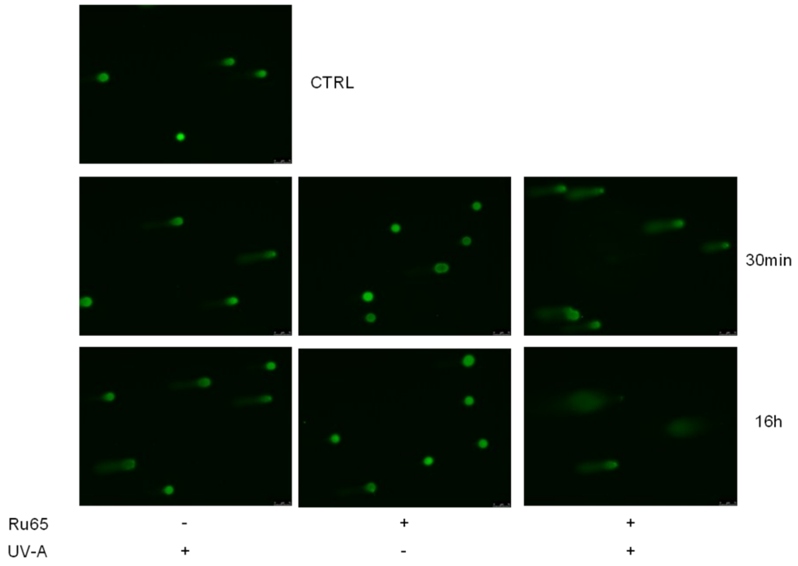

Figure S3 - Alkaline comet assay (related to Fig. 3)

Representative micrographs of fluorescent DNA stain in U2OS cells that were treated as indicated and examined by alkaline comet assay.

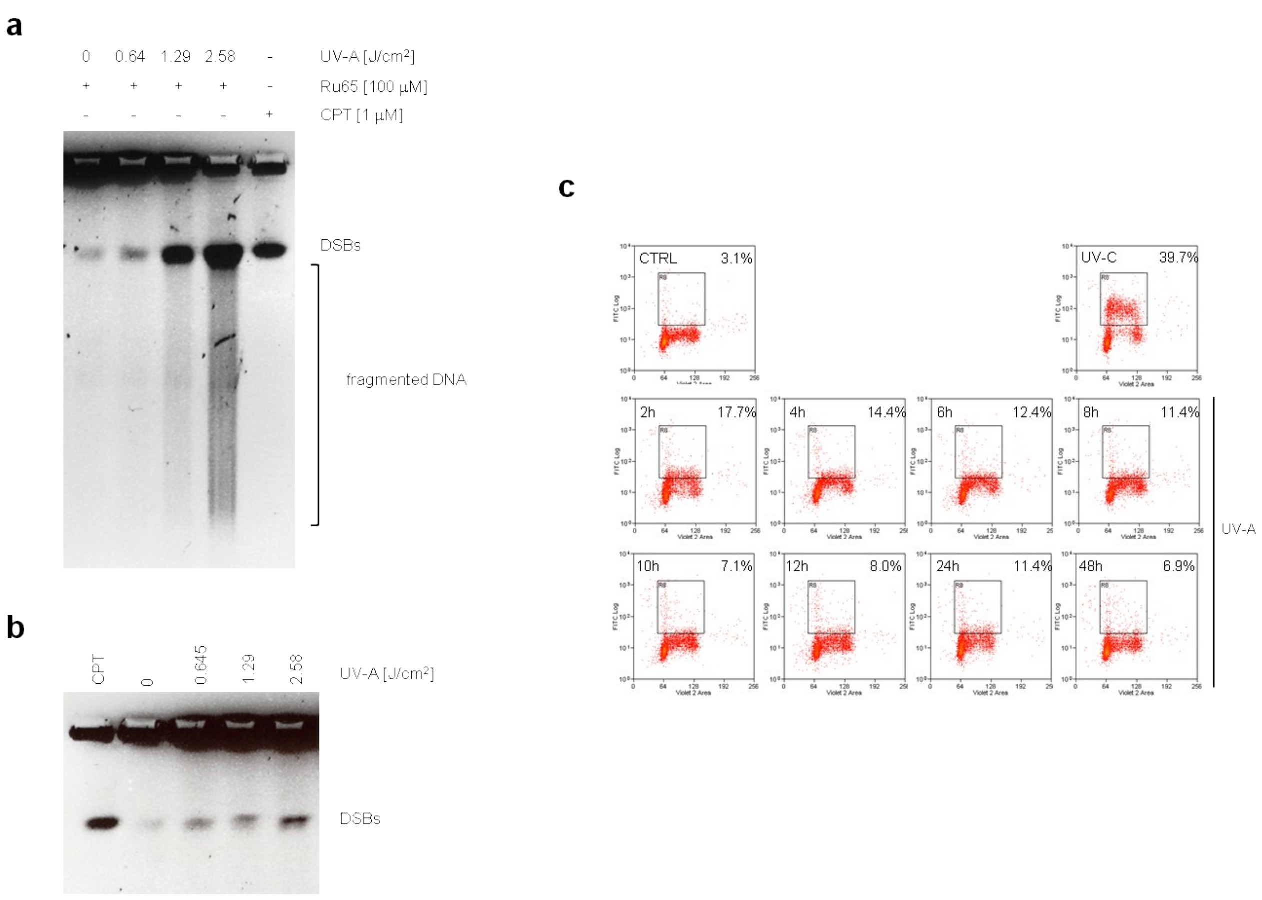

Figure S4 – Effect of the photo-effector UV-A (related to Fig. 3)

a. PFGE analysis of U2OS cells treated with the indicated amounts of Ru65 and increasing doses of UV-A. Camptothecin (CPT 1 µM, 4h) was used as positive control.

b. PFGE analysis of U2OS cells treated with the indicated amounts of UV-A. Camptothecin (CPT 1 µM, 4h) was used as positive control.

c. Time-course flow cytometric analysis of H2AX phosphorylation in U2OS cells treated with UV-A (1.29 J/cm^2). Irradiation with UV-C (20 J/m^2) was used as positive control.

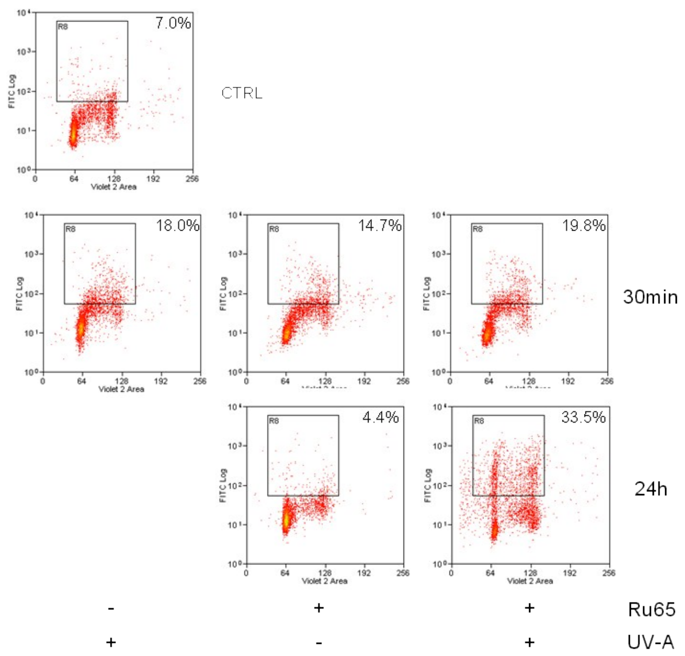

Figure S5 – Response of head and neck carcinoma cells to photo-irradiation of Ru65 (related to Fig. 3)

CAL33 cells were treated with Ru65 (50 µM) and UV-A (1.29 J/cm^2) as indicated. Upon fixation, cells were probed with anti-γH2AX antibody and examined by flow cytometry.

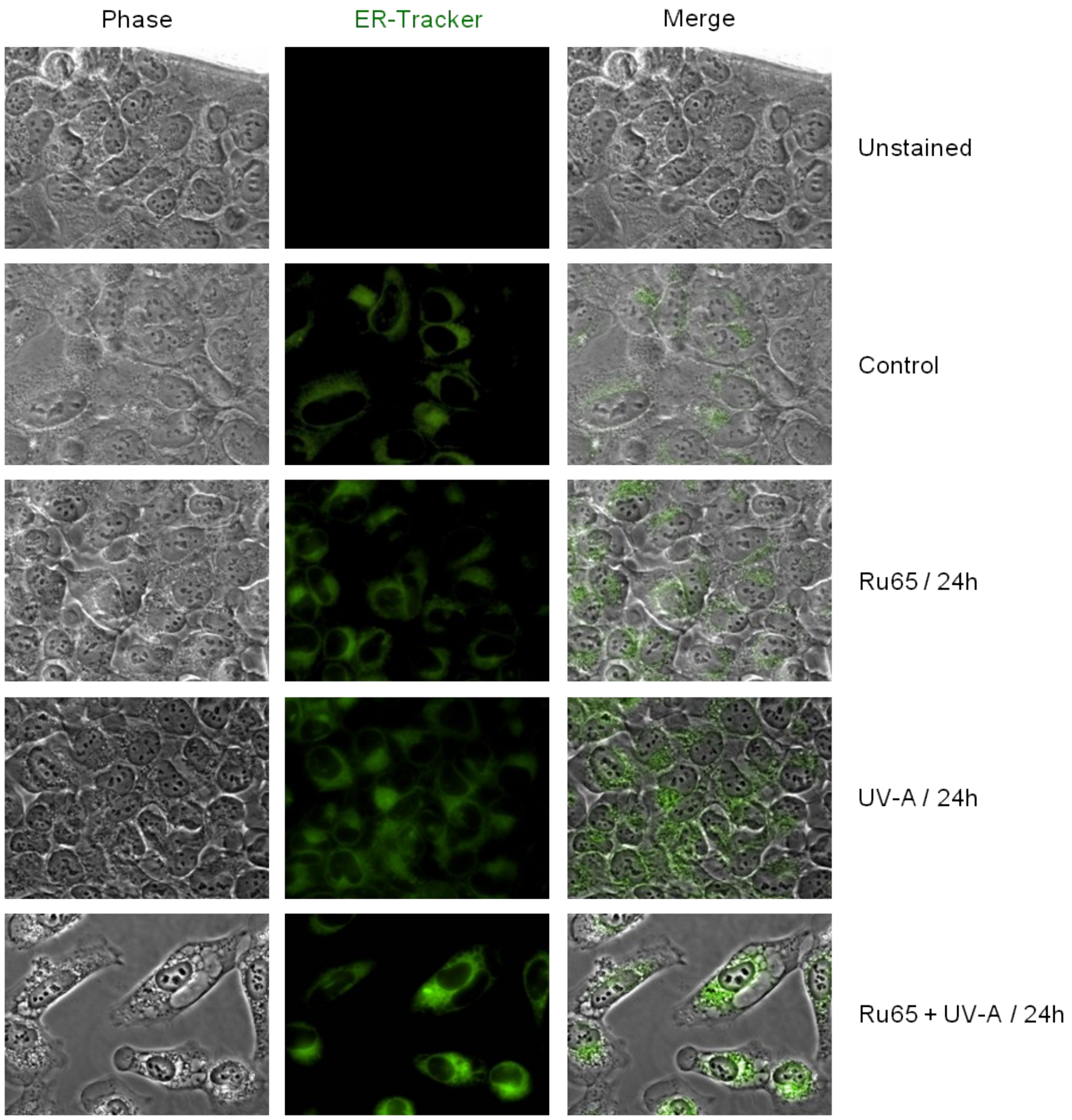

Figure S6 – Photo-irradiation of Ru65 causes an ER-dependent stress response (related to Fig. 4)

Phase contrast, endoplasmic reticulum (ER) staining (fluorescence) and merged images of U2OS cells left untreated or treated as indicated.

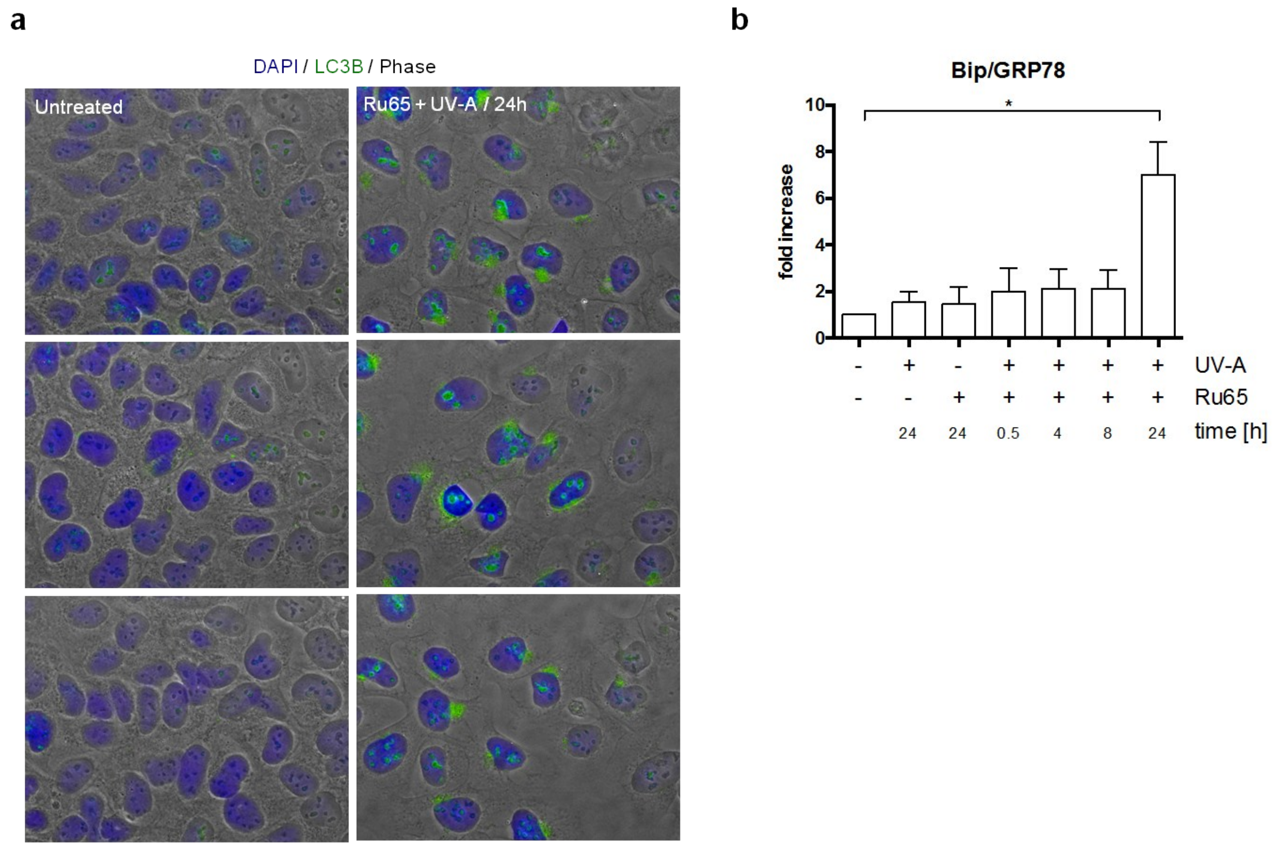

Figure S7 – Detection and quantification of ER-stress response markers upon photo-irradiation of Ru65 (related to Fig. 4)

a. Merged phase contrast and immunofluorescence images of LC3B expression in U2OS cells treated as indicated.
b. Quantification of the signal corresponding to BIP/GRP78 expression was obtained using ImageJ and normalized to the unsaturated signals of α-tubulin. The fold increase was obtained by normalizing each treatment to the untreated control. Statistical analysis was performed using GraphPad Prism software by unpaired t-test. Values are presented as mean ± SEM. $n = 2$ different experiments. *$p < 0.05$.

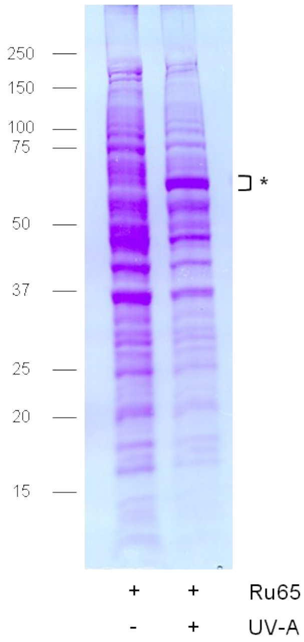

Figure S8 – Protein expression pattern upon photo-irradiation of Ru65 (related to Fig. 4)

Coomassie blue-stained whole cell extracts obtained from U2OS cells treated as indicated. Proteins present in the band indicated with an asterisk were submitted to mass spectrometry.

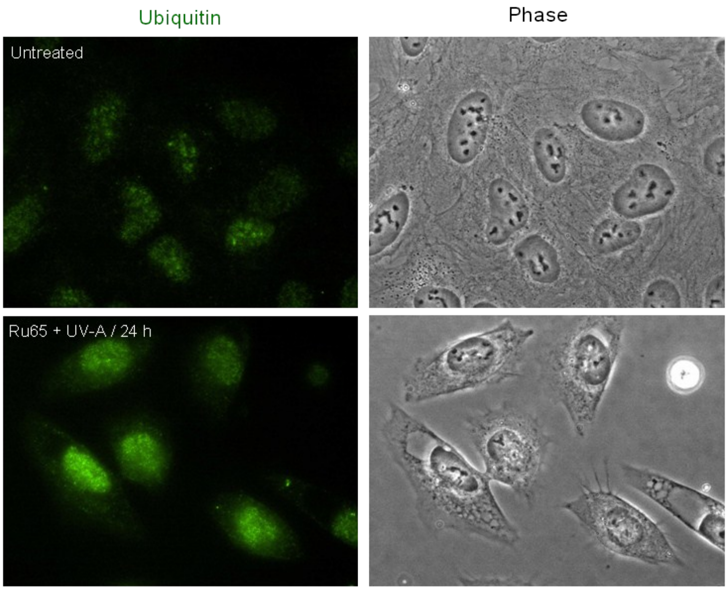

Figure S9 – Detection and quantification of ER-stress response markers upon photo-irradiation of Ru65 (related to Fig. 4)

Entire field immunofluorescence and phase contrast images of protein ubiquitylation in U2OS cells treated with Ru65.

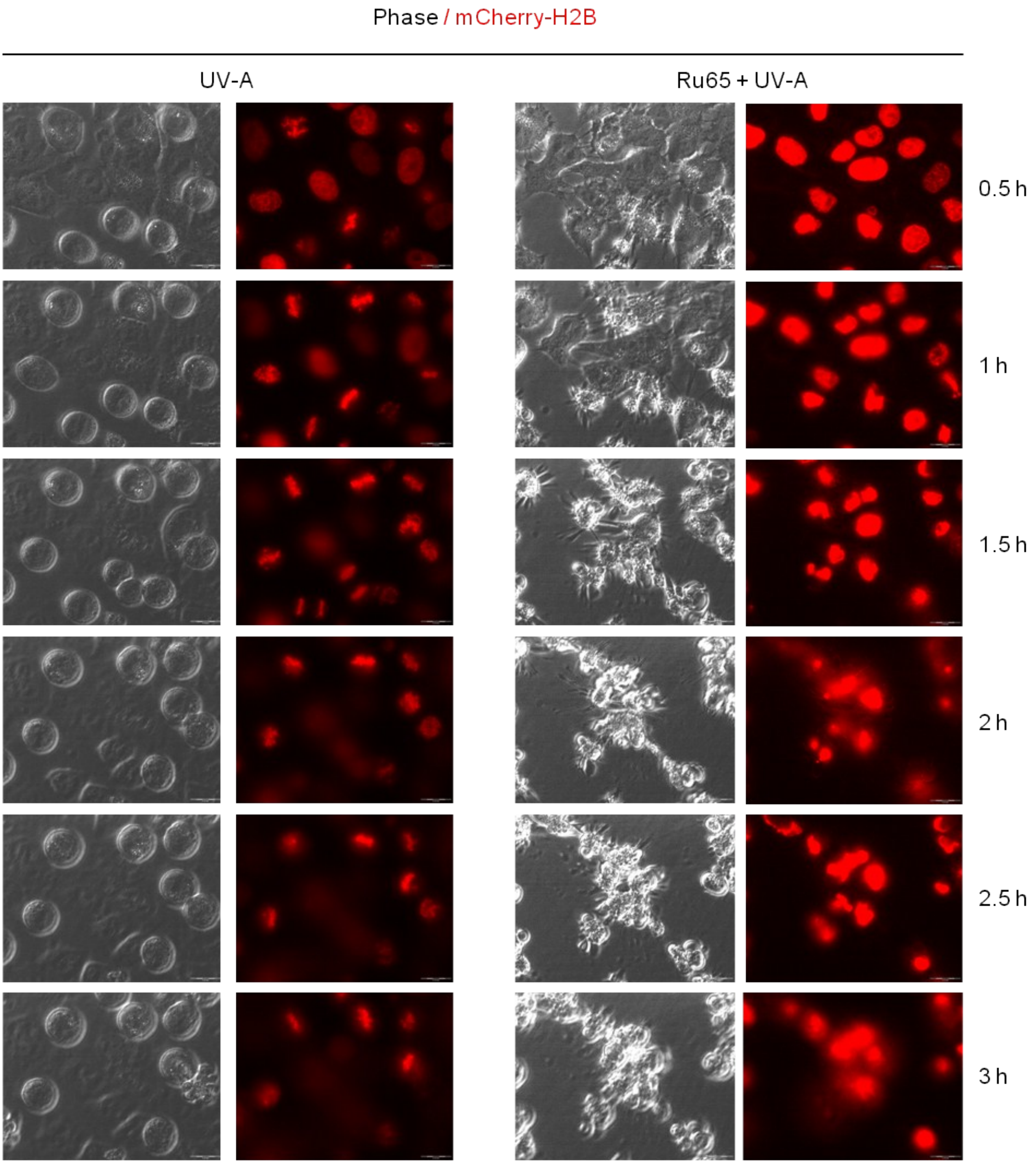

Figure S10 – Effect of Ru65 photo-irradiation on the entry into mitosis (related to Fig. 5)

Phase contrast and immunofluorescence stills of transition through mitosis (time-course) upon release from RO-3306 and photo-irradiation of Ru65.

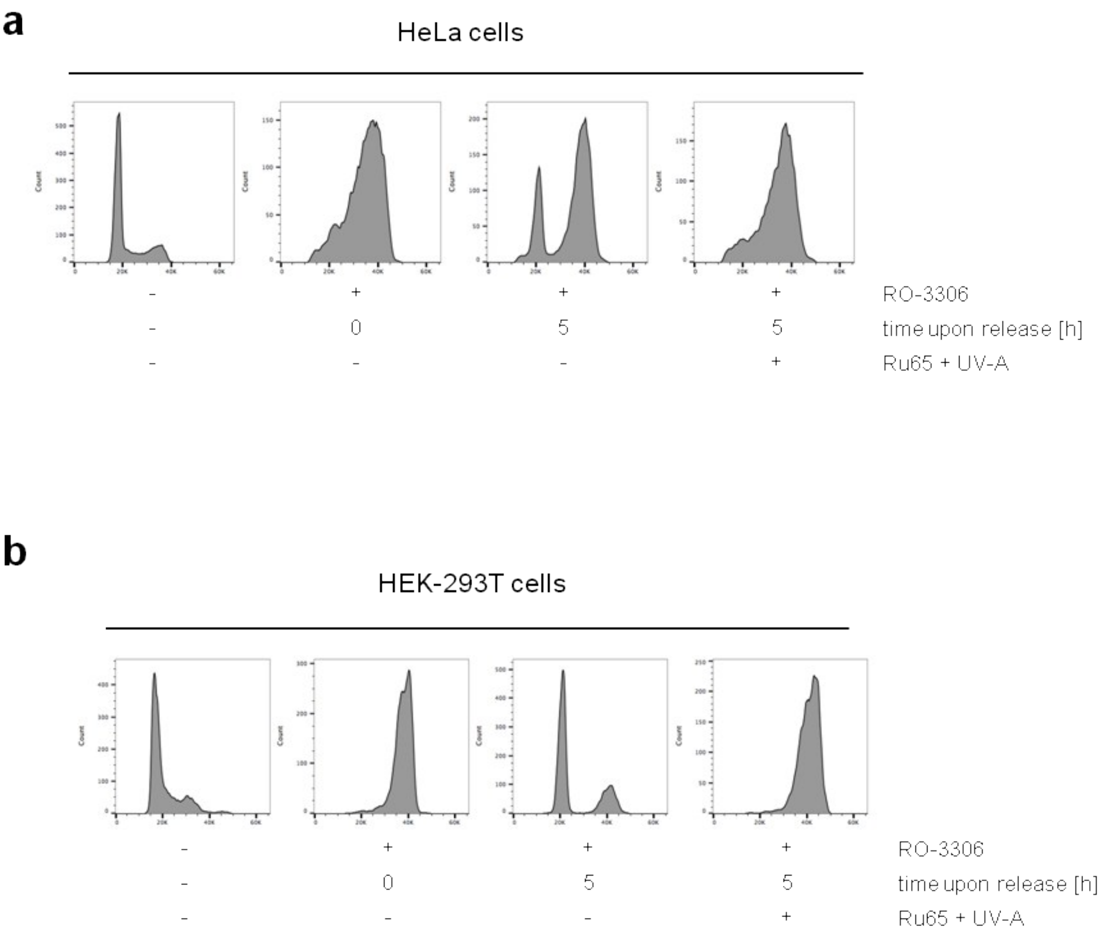

Figure S11 – Effect ofRu-65 photo-irradiation on cell cycle progression(related to Fig. 5)

a. HeLa cells were synchronized with RO-3306 (9 µM, 15h) and treated with Ru65 (50 µM) for the last 2h of incubation. Cells were irradiated with UV-A (1.29 J/cm^2) at the time of release from RO-3306. Cells were fixed, stained with DAPI and the DNA content was examined by flow cytometry.
b. HEK-293T cells were treated and examined as in a.

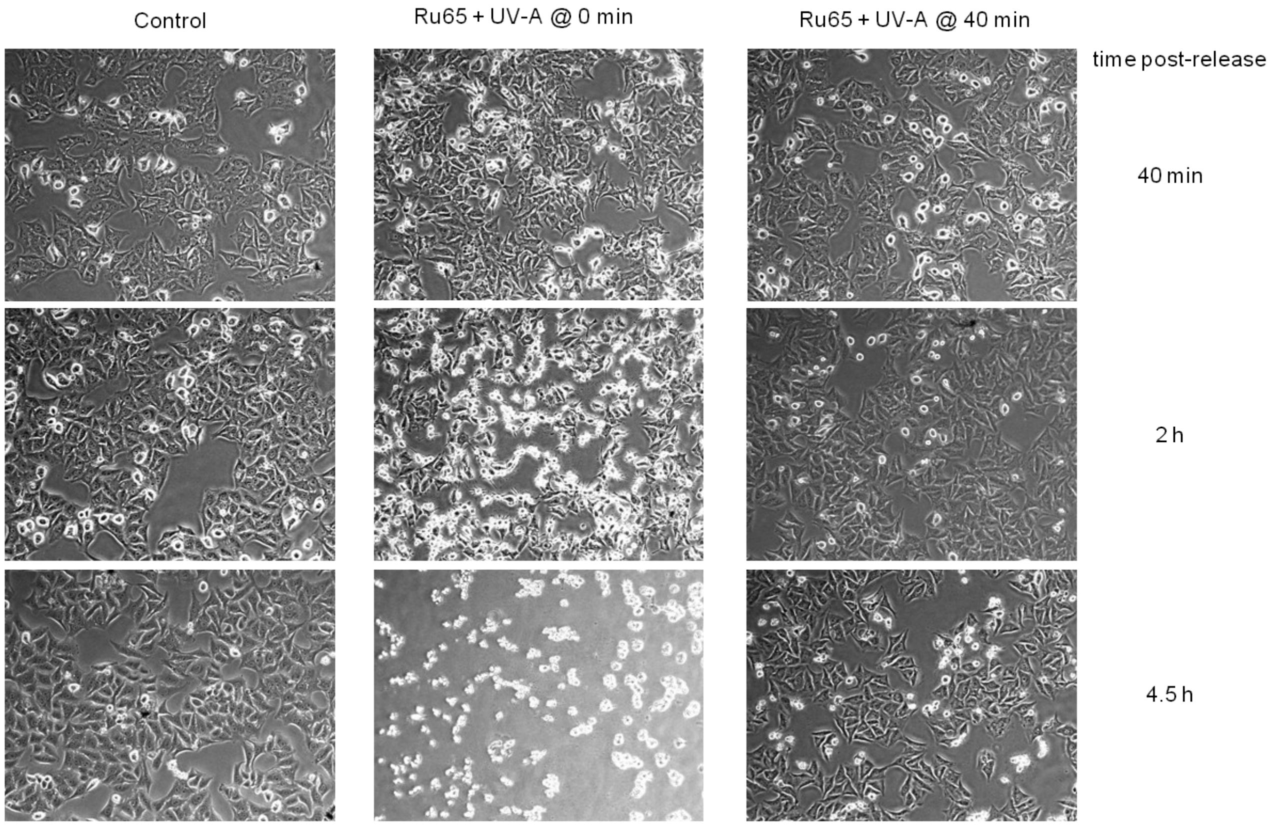

Figure S12 – Effect of Ru65 photo-irradiation on cells in advanced mitosis (related to Fig. 5)

Phase contrast stills of cells that were treated with RO-3306 (9 µM) for 15 h and Ru65 (100 µM) for the last 2h of incubation. Cells were irradiated with UV-A (1.29 J/cm^2) at the time of release from RO-3306 (middle panels) or 40 min upon release from RO-3306 (right panels) and visualized at the indicated time points.

Supplementary Movies

Movie S1 – Time-course visualization of cytoplasmic vacuole formation

Time-course cytoplasmic vacuole formation in U2OS cells treated with Ru65. The complex was activated with UV-A (1.29 J/cm^2) and the process of vacuole formation was documented acquiring four frames per hour over a period of 16h.

Movie S2 – Time-course visualization of mitotic transition in synchronized cells

Merge of phase contrast and fluorescence frames visualizing RO-3306-synchronized HeLa mCherry-H2B cells that were UV-A irradiated at the time of release from RO-3306. Twelve frames per hour were acquired over a period of 3h.

Movie S3 – Time-course visualization of mitotic transition in synchronized cells treated with Ru65

Merge of phase contrast and fluorescence frames visualizing RO-3306-synchronized HeLa mCherry-H2B cells that underwent Ru65 photo-activation at the time of release from RO-3306. Twelve frames per hour were acquired over a period of 3h.

REFERENCES

1. L. T. Vassilev, C. Tovar, S. Chen, D. Knezevic, X. Zhao, H. Sun, D. C. Heimbrook and L. Chen, *Proc Natl Acad Sci U S A*, 2006, **103**, 10660-10665.
2. G. Pratviel and B. Meunier, *Chemistry*, 2006, **12**, 6018-6030.
3. C. Mari, V. Pierroz, R. Rubbiani, M. Patra, J. Hess, B. Spingler, L. Oehninger, J. Schur, I. Ott, L. Salassa, S. Ferrari and G. Gasser, *Chem. Eur. J.*, 2014, **20**, 14421-14436.
4. A. Frei, R. Rubbiani, S. Tubafard, O. Blacque, P. Anstaett, A. Felgentrager, T. Maisch, L. Spiccia and G. Gasser, *J Med Chem*, 2014, **57**, 7280-7292.
5. T. Mori, M. Nakane, T. Hattori, T. Matsunaga, M. Ihara and O. Nikaido, *Photochem Photobiol*, 1991, **54**, 225-232.
6. M. El-Shemerly, D. Hess, A. K. Pyakurel, S. Moselhy and S. Ferrari, *Nucleic Acids Res*, 2008, **36**, 511-519.
7. R. Rubbiani, S. Can, I. Kitanovic, H. Alborzinia, M. Stefanopoulou, M. Kokoschka, S. Monchgesang, W. S. Sheldrick, S. Wolfl and I. Ott, *J Med Chem*, 2011, **54**, 8646-8657.
8. A. M. Rieger, K. L. Nelson, J. D. Konowalchuk and D. R. Barreda, *J Vis Exp*, 2011, DOI: 10.3791/2597.
